# High Troponin-T in Acute Biliary Pancreatitis: Is it a Real Myocardial Injury?

**DOI:** 10.7759/cureus.18637

**Published:** 2021-10-10

**Authors:** Hany A Zaki, Eman E Shaban, Ahmed E Shaban, Amr Elmoheen

**Affiliations:** 1 Emergency Medicine, Hamad Medical Corporation, Doha, QAT; 2 Cardiology, Aljufairi Diagnostic and Therapeutic Hospital, Doha, QAT; 3 Internal Medicine, Mansoura General Hospital, Mansoura, EGY

**Keywords:** lipase, elevated amylase, elevated liver enzyme, cardiac markers, myocardial infarction, high troponin, acute biliary pancreatitis

## Abstract

Acute pancreatitis (AP) is characterized by abdominal pain and elevated levels of pancreatic enzymes in the serum. Pain is the hallmark of this condition, and as a presenting symptom, is localized in the epigastrium in at least 60% of patients having the mild or severe form of the disease. Thus, the differential diagnosis may be difficult in some cases due in part to the fact that the disease may mimic other diseases, and in particular, acute coronary syndrome. We present the case of a patient who presented to our facility with epigastric pain, normal electrocardiogram (ECG), elevated high-sensitive troponin-T and elevated lipase, and amylase. Laboratory investigations and ultrasonography confirmed AP, with further serial ECGs being within the normal limits and normal echocardiography. The patient underwent laparoscopic cholecystectomy and intraoperative cholangiogram. Postoperative diagnosis confirmed biliary pancreatitis with chronic cholecystitis.

## Introduction

Acute pancreatitis (AP), as the name implies, is a sudden inflammation of the pancreas. Major characteristics include sudden abdominal pain and high serum levels of pancreatic enzymes [1]. The incidence of this disorder ranges from 10 to 50/100,000 per annum [2]. It has an overall mortality rate of 4%-6%, with the mortality increasing to 17%-39% in extreme cases [3].

Gallstones are the major cause of pancreatitis in first-world countries. Studies have shown that it accounts for over 60% of cases [4-5]. Studies have shown that biliary pancreatitis is as common as alcoholic pancreatitis, accounting for no less than 66% of cases [6]. Biliary (gallstone) pancreatitis can be diagnosed via ultrasonography and laboratory tests (liver enzymes) [7-8]. Ultrasonography has >95% sensitivity in detecting gallstones in uncomplicated cases. However, this sensitivity reduces to 67%-68% when ileus is affected [7]. A triple-fold elevation of alanine aminotransferase has a 95% positive predictive value in the detection of gallstones as a cause of pancreatitis [8]. Endoluminal ultrasonography and magnetic resonance cholangiopancreatography (MRCP) may be used as additional diagnostic tools, although their role in biliary pancreatitis has not yet been evaluated [3].

Most biliary pancreatitis patients recover without a significant sequela. But then, over 30% of cases are characterized by severe episodes requiring multidisciplinary care [9]. Complications commonly associated with this disorder are local (abscesses, formation of pseudocyst, necrosis, hemorrhage) and systemic (adult respiratory distress syndrome, pleural effusion, multiorgan failure, and renal insufficiency) [9-10].

As established, most cases of pancreatitis are observed in alcoholics. However, the evidence of chest pain together with positive troponin-I and little to no electrocardiogram changes in the setting of pancreatitis can cause complications in the management of the disease, thus initiating a controversy on whether to start medical treatment of acute myocardial infarction (antiplatelets and anticoagulants) or not. We understand that no such cases have been reported in the medical literature of AP with non-ST-segment elevation myocardial infarction. Elevated troponin levels without acute coronary syndrome are mostly caused by myocyte necrosis, primarily due to a mismatch between the supply and demand of oxygen due to abnormal loading of both sides of the heart (acute or chronic) [11-12]. It is important to note that there is always an increase in troponin with tachycardia, after infusion or release of tachycardia, after strenuous exercise, imbalance of the autonomic nervous system, and in conditions that have a direct effect on membrane permeability [11]. This article discusses the management of a patient who similarly presented with pancreatitis with high troponin levels and the outcome.

## Case presentation

A 38-year-old male patient who came to our emergency department (ED) presents with sudden onset epigastric pain around 9:30 pm while driving. The pain was so severe; the patient described feeling that he was going to die. He felt dizzy and about to lose his consciousness, was not able to control his vehicle, which resulted in a road traffic accident. The pain lasted around 30 minutes and was associated with profuse sweating and nausea, during which he vomited twice in the ED. There was no shortness of breath. A similar attack that he had four months ago had been attributed to Helicobacter pylori. He had no significant past medical history and was a non-smoker. General examination showed the patient appeared well but anxious and in pain, apyrexial. His vital data were temperature 37.60°C (oral), respiratory rate 16 breaths per minute, blood pressure 90/65 mmHg, and O2 saturation 97% on room air.

Physical examination of the patient yielded that he was conscious, alert, and had no neurological manifestation. His chest examination showed normal chest expansion, and breath sounds were equal bilaterally with no added sounds. His heart sounds were normal, with no audible murmurs. He had normal jugular venous pressure and no pedal edema. His abdomen was not distended and not rigid, but he had tenderness at the epigastric area with mild guarding. He had intact hernial orifices and normal bowel sounds.

Investigations

**Figure 1:**
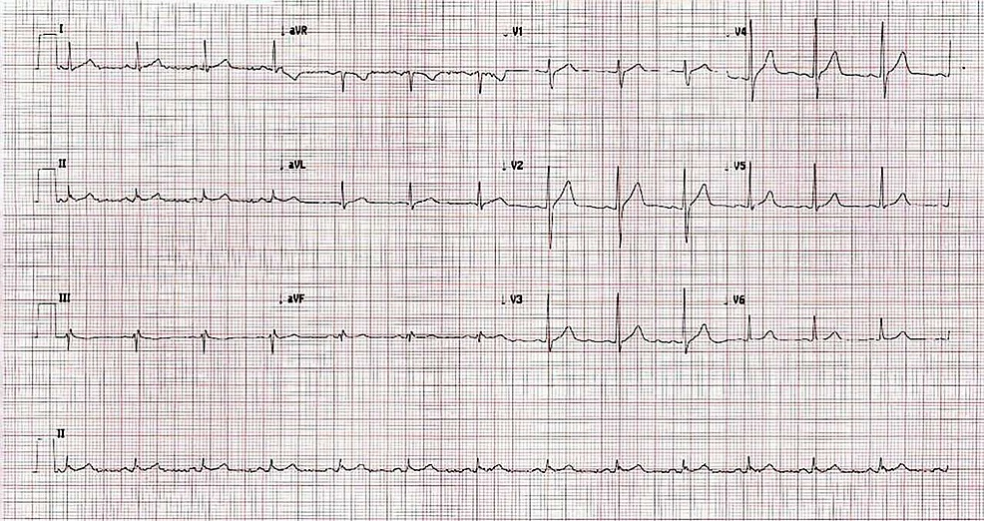
ECG: sinus rhythm and no signs of acute cardiac ischemia. ECG, electrocardiogram

**Figure 2:**
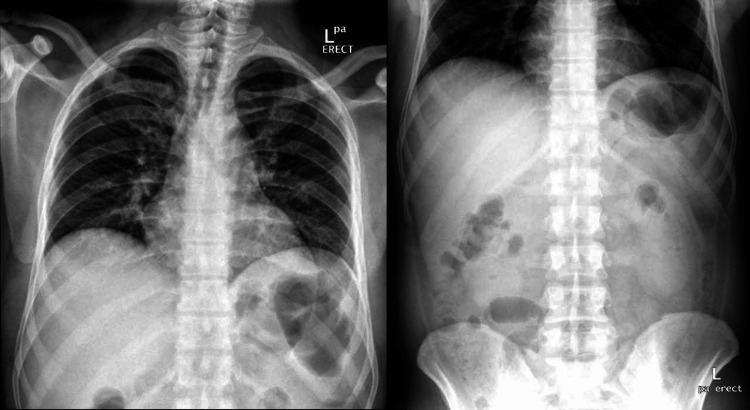
Chest X-ray, erect position (left): normal chest X-ray, no signs of pneumonia, or air-under diaphragm. Abdominal X-ray, erect position (right): normal bowel gas pattern. No evidence of bowel wall thickening. The vertebral column appears normal.

**Figure 3:**
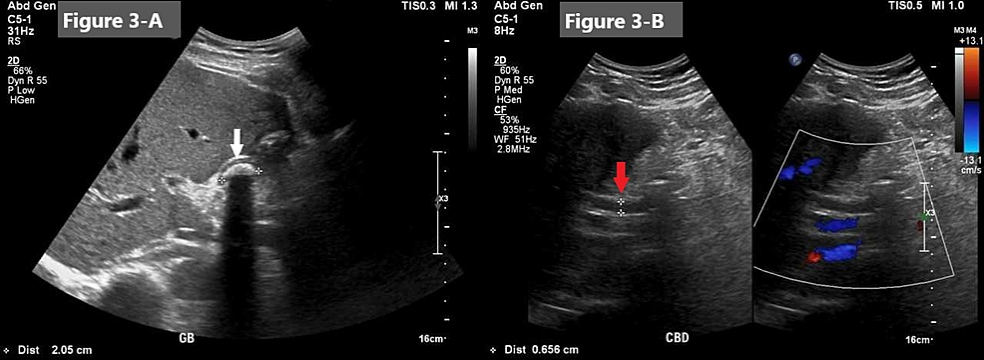
Ultrasound image (A, left): the gallbladder was contracted and demonstrated a 20 mm calculus (white arrow). (B, right): CBD was normal in caliber, measuring 5-6 mm (red arrow). CBD, common bile duct

His electrocardiogram (ECG) showed sinus rhythm and no signs of acute cardiac ischemia (Figure 1). The chest and abdomen X-rays did not show pneumonia or air under the diaphragm (Figure 2). His laboratory reports showed a highly elevated troponin-T, amylase, and lipase (Table 1). Ultrasound abdomen was performed and showed the gallbladder was contracted and demonstrated a 20 mm calculus. The common bile duct (CBD) was normal in caliber, measuring 5-6 mm (Figure 3). Echocardiography was done and showed normal global systolic left ventricular function, and ejection fraction (EF) was 55%. Echocardiography did not demonstrate any regional wall motion abnormality, and there was evidence of atrial septal aneurysm (Video 1).

**Video 1:** Echocardiography (parasternal long-axis, parasternal short-axis, apical, and two chamber views): normal global systolic left ventricular function, and EF was 55%. Echocardiography did not demonstrate any regional wall motion abnormality, and there was evidence of atrial septal aneurysm. EF, ejection fraction

**Table 1:** Laboratory investigation results, showing a significant rise of troponin-T, pancreatic amylase, lipase, and normal troponin-I. ALT, alanine transaminase

Test	Result	Normal range
Total bilirubin	69.0 µmol/L	3.4-20.5 µmol/L
ALT	214 U/L	0-55 U/L
Pancreatic amylase	939 U/L	8-51 U/L
Lipase	3,117 U/L	8-78 U/L
Lactic acid	2.1 mmol/L	0.5-.6 mmol/L
Troponin-T	4,600.0 ng/L	0.0-17.0 ng/L
Troponin-I	<0.3 ng/mL	0.0-0.3 ng/mL

Differential diagnosis

The patient presented to the ED with severe epigastric pain, sweating, and near syncope. Although he had a normal ECG, he had highly elevated troponin-T, which raised the probability of the diagnosis of acute coronary syndrome. The echocardiography performed did not show evidence of regional wall motion abnormality. Troponin-I was done and was normal (<0.3 ng/mL), which ruled out myocardial injury. On the other hand, the ultrasound abdomen showed evidence of gall bladder stone, and the laboratory results showed highly elevated bilirubin, amylase, and lipase. Hence, the diagnosis of biliary pancreatitis was confirmed.

Treatment

The patient was admitted to the ward of general surgery and received IV fluids and analgesics. He underwent laparoscopic cholecystectomy and intraoperative cholangiogram (IOC) with the retrieval of common bile duct stones by Dormia basket. He had a smooth operation, and he did not develop any acute complications. The patient was discharged home after five days.

Outcome and follow-up

The patient improved, and the pain ultimately subsided. He followed with the gastroenterology and general surgery outpatient clinic several times until he returned to his everyday life. The patient has been referred to a cardiology outpatient clinic, and the cardiologist advised for further imaging such as CT coronary angiogram or percutaneous coronary intervention (PCI). Still, the patient was not obedient to do so.

## Discussion

Cardiac troponin is unique to muscles of the heart and is reliable for the detection of injury to the heart muscle [13]; however, they attain a peak level as early as eight hours after myocardial injury. Thus, we did the assay eight hours after the onset of chest pain. There have been previous reports of AP with changes in ECG [14-15]. Elevation of troponin is an indication of injury to the myocardial cells, which may be triggered by trauma, inflammation, ischemia, tachycardia, strenuous exercise, infusion or release of catecholamines, imbalance in the autonomic nervous system, conditions that have a direct effect on membrane permeability, chest contusion, toxins, systemic infection, electricity infiltrative disease, or renal failure. We do not have a full understanding of the mechanism involved in troponin release in pancreatitis. We know that pancreatic enzymes travel through tissue planes destroying tissues and formation of pseudocysts at locations that are distant to the pancreas, a theory similar to diaphragmatic movement may also hold for direct myocardial injury. Also, animal studies have suggested that pancreatic enzymes may enter the general circulation and cause myocardial injury. Coronary vasospasm or changes in the permeability of the myocardial cell membrane secondary to AP may also be a possibility. Microvascular dysfunction due to pancreatitis may result in myocardial stunning [16].

Complications due to AP involving true or pseudo myocardial infarction are rare [17]. There has been previous documentation of ECG changes bearing close resemblance to acute myocardial infarction in AP patients [17]. However, to the best of our knowledge, there has been no documentation of any occurrence of non-ST-segment elevation myocardial infarction in AP. Thrombolysis of a pancreatitis patient in such a case may be fatal. A report by Main et al. illustrated a case of a 47-year-old male who suffered alcohol-related AP. The patient in Main’s case died of severe retroperitoneal hematoma due to thrombolytic therapy [18]. Therefore, there is a need for a standard management protocol to be set for the management of patients in a similar condition. Intensive interventions should be taken into consideration when such complicated cases present, and in case of doubt, the findings need to be verified with a coronary angiogram.

Serum markers are required for the rapid identification of patients with cardiac involvement during AP.

In clinical settings, elevated troponin levels have been found in AP patients even in the absence of acute myocardial damage. In a study conducted recently, we evaluated the presence of increased high-sensitivity cardiac troponin (hs-TnT) in AP patients [19]. We discovered that at least 35% of the patients had elevated serum levels of this marker. However, because there were no electrocardiographic or clinical features of acute coronary syndrome in our patient, it serves as a pointer that abnormally high results should be considered as a potential presence of rhabdomyolysis and not acute cardiac injury.

In a study examining myoglobin in patients with AP [20], we discovered that 20% of patients with AP had elevated myoglobin concentrations in the serum while patients with mild cases of pancreatitis had myoglobin concentrations similar to patients with severe pancreatitis.

## Conclusions

The mechanism of troponin release in AP is not fully understood. There are infrequent occurrences of complications of AP involving true or pseudo myocardial infarction. Studies have shown that troponin-I has the potential to classify rhabdomyolysis patients accurately, as compared to troponin-T. Troponin-I should be used in the assessment of cardiac damage in patients with AP. However, there is a need for further studies to explore this possibility. Further imaging or intervention after recovery from the acute illness may be needed for the possibility of coronary pathology.
